# Secondary Nucleation and the Conservation of Structural Characteristics of Amyloid Fibril Strains

**DOI:** 10.3389/fmolb.2021.669994

**Published:** 2021-04-16

**Authors:** Saeid Hadi Alijanvand, Alessia Peduzzo, Alexander K. Buell

**Affiliations:** ^1^Bioprocess Engineering Department, Institute of Industrial and Environmental Biotechnology, National Institute of Genetic Engineering and Biotechnology, Tehran, Iran; ^2^Technical University of Denmark, Department of Biotechnology and Biomedicine, Lyngby, Denmark

**Keywords:** amyloid, secondary nucleation, α-synuclein, amyloid beta, proliferation

## Abstract

Amyloid fibrils are ordered protein aggregates and a hallmark of many severe neurodegenerative diseases. Amyloid fibrils form through primary nucleation from monomeric protein, grow through monomer addition and proliferate through fragmentation or through the nucleation of new fibrils on the surface of existing fibrils (secondary nucleation). It is currently still unclear how amyloid fibrils initially form in the brain of affected individuals and how they are amplified. A given amyloid protein can sometimes form fibrils of different structure under different solution conditions *in vitro*, but often fibrils found in patients are highly homogeneous. These findings suggest that the processes that amplify amyloid fibrils *in vivo* can in some cases preserve the structural characteristics of the initial seed fibrils. It has been known for many years that fibril growth by monomer addition maintains the structure of the seed fibril, as the latter acts as a template that imposes its fold on the newly added monomer. However, for fibrils that are formed through secondary nucleation it was, until recently, not clear whether the structure of the seed fibril is preserved. Here we review the experimental evidence on this question that has emerged over the last years. The overall picture is that the fibril strain that forms through secondary nucleation is mostly defined by the solution conditions and intrinsic structural preferences, and not by the seed fibril strain.

## Introduction

Amyloid fibrils are highly ordered protein polymers and represent a common hallmark of a range of severe human disorders, many of which are neurodegenerative in nature (Knowles et al., 2014). Particularly prominent examples include Alzheimer’s disease, Parkinson’s disease, amyotrophic lateral sclerosis (ALS) and Creutzfeldt-Jakob disease. The most striking common feature of these diseases is their progressive nature, whereby the amyloid fibril pathology involves ever increasing regions of the central nervous system (CNS), as the disease advances (Braak and Braak, 1991; Brettschneider et al., 2015). It was found that often the pathology originates in a localised area of the nervous system, from which it spreads for up to several decades through a well-defined pathway toward other regions until the overall damage to the nervous system is fatal.

The quantity of fibrillar material inside a patient increases over time, and this increase appears to proceed along a defined, connected trajectory. These observations suggest that processes are at work that amplify the low quantities of amyloid fibrils that are present in the very early, asymptomatic stages of the disease. The amyloid proteins and peptides (e.g., α-synuclein, Aβ) are often found throughout most of the brain in their monomeric forms, but new fibrils do not seem to form independently in different parts of the brain. Therefore, the presence of fibrils at a given location facilitates the formation of more fibrils, ultimately leading to spreading. Fibril amplification can either proceed through repeated cycles of fragmentation and elongation, or else through secondary nucleation. Combined growth and fragmentation has been shown in many studies to allow efficient amplification of very small quantities of fibrils for diagnostic or analytical purposes *in vitro*, e.g., in prion misfolding cyclic amplification (PMCA; Saá et al., 2006) or in real-time quaking-induced conversion (RT-QuIC; Saijo et al., 2019). Secondary nucleation, on the other hand, corresponds to the nucleation of new fibrils from monomer that is facilitated by the presence of existing fibrils, e.g., through surface-catalysis (heterogeneous nucleation; Buell, 2017)). One of the key questions that remains to be answered in the field of protein aggregation and misfolding diseases is as to which of these, if any, amplification mechanisms plays the dominant role in the spreading of a particular amyloid pathology. Such knowledge would enable a targeted intervention, as there is good theoretical evidence that interference with the amplification mechanism could be the most effective therapeutic strategy (Michaels et al., 2019).

Evidence that secondary nucleation might play a role in fibril amplification *in vivo* comes from *in vitro* experiments, in which secondary nucleation rates can be very high even under completely quiescent conditions (Cohen et al., 2013; Buell et al., 2014). On the other hand, fibril fragmentation rates have been shown to be very strongly increased by mechanical agitation or stirring (Xue and Radford, 2013), conditions which are unlikely to be replicated *in vivo.* Indeed, it is experimentally challenging to detect fibril fragmentation under completely quiescent conditions, and the little available data suggests very low rates (Smith et al., 2006; Fränzl et al., 2019).

In this review, we will focus on another line of available experimental evidence, namely the propagation of structural properties. Amyloid structural biology has experienced a breakthrough in recent years, due to the availability of atomic resolution structures of amyloid fibrils from cryo-electron (cryo-EM) microscopy. This method is applicable both to fibrils formed *in vitro* (Gremer et al., 2017; Guerrero-Ferreira et al., 2018; Röder et al., 2019), as well as to fibrils derived from patients [Aβ (Kollmer et al., 2019), α-synuclein (Schweighauser et al., 2020), tau (Fitzpatrick et al., 2017) and immunoglobulin light chains (Radamaker et al., 2019)].

Lower resolution structural and biochemical information had been available for years and had suggested that a given amino acid sequence is able to form different types of amyloid fibrils, called strains, often under different solution conditions (Toyama et al., 2007). The existence of such fibril strains has now been given a firm structural basis through high resolution cryo-EM structures (Li et al., 2018).

It is found that patient-derived amyloid fibrils are often very homogeneous (Cendrowska et al., 2020) and consist of a small number of polymorphs (Fitzpatrick et al., 2017; Schweighauser et al., 2020). Different patients with the same amyloid disease (Annamalai et al., 2016), as well as patients with different diseases, but involving the same protein (Schweighauser et al., 2020), can display different fibril structures. However, there are also cases where patient-derived fibrils have been found to show a higher degree of polymorphism (Kollmer et al., 2019).

The fact that fibrils extracted from a given patient are of the same, or a small number of, morphologies suggests that the molecular processes at work to amplify the fibrils can at least in some cases preserve the structural information encoded in the initial seed fibrils. An alternative explanation is that the specific physico-chemical environment in the given patient is only conducive to the formation of very specific type(s) of fibrils. An answer to the question whether or not secondary nucleation is able to preserve the structural information of the seed fibril is therefore a pertinent one in this context (Linse, 2017), and we will review here the available evidence. Before reviewing secondary nucleation, we will summarize the available evidence for the preservation of the fibril strain by fibril elongation/growth.

## Fibril Elongation Preserves the Strain Characteristics

**FIGURE 1:**
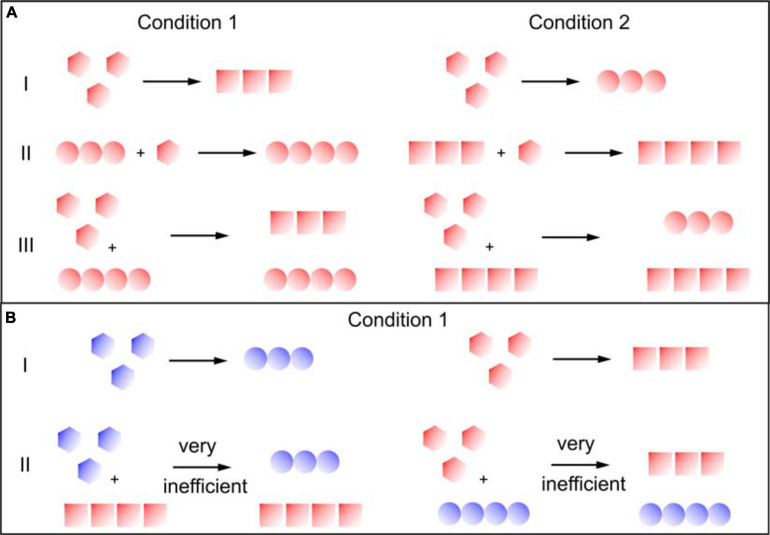
Secondary nucleation and the transmission of structural information. Different colors indicate different sequences and different symbols indicate different structures. **(A)** Proteins where the same sequence can form different strains under different conditions (e.g., insulin, α-synuclein). I: Fibrils formed through primary nucleation from monomer alone form different fibril strains, depending on the solution conditions. II: In fibril elongation, the templating effect of the seed fibrils guides the monomer into a conformation that is not dictated by the solution conditions, but by the seed. III: in secondary nucleation, the newly formed fibril strains are defined by the solution conditions, rather than the seed fibrils. **(B)** Proteins where closely related sequences have different structural preferences (e.g., Aβ 1-40 and Aβ 1-42). I: the related sequences may form different fibrils even under identical solution conditions. II: Cross-surface nucleation can be inefficient if the monomers have a different structural preference from the seed fibrils.

Fibril strain propagation is the capability of preserving the physical and structural properties of the seed fibrils even under conditions that favour the *de novo* nucleation of another fibril structure (Figure 1A). In this process, the seed can act as a template and transmit its conformational properties to the incorporating monomer by “conformational memory” (Yamaguchi et al., 2005). Seeding is very efficient in accelerating the aggregation reaction, as the energy barrier for the primary nucleation of new fibrils from monomer is significantly higher than that for the incorporation of monomers at the end of the existing fibril, even if this addition does not lead to the formation of the most stable state under the particular set of solution conditions (Buell, 2019). The reason for which seeding is so effective, as well as the mechanism by which the seed fibril guides the incoming monomer into the template structure, are not known in detail. However, the fact that fibril elongation rates saturate at high monomer concentration (Buell, 2019) indicates that elongation involves more than a single molecular step. It probably involves an initial weak association between the monomer and the seed fibril end, followed by a conformational search for the thermodynamically most stable state (Jia et al., 2020). Sometimes the fibril end can remain in a state in which the last incoming monomer has not yet reached the correct structure for extended periods of time, as suggested by the fibril’s inability to grow further during this time period, i.e., stop-and-go-kinetics of fibril growth (Ferkinghoff-Borg et al., 2010). There is also increasing evidence that the transition state for fibril elongation, i.e., the highest free energy state separating the isolated monomer from the fully incorporated one, involves a close contact between the fibril and the monomer (Vettore and Buell, 2019). Overall, there is strong evidence that fibril growth mechanisms correspond to induced fit, rather than conformational selection (Arai et al., 2015). The latter would correspond to the fibril end “fishing” the appropriate pre-formed monomer conformation out of solution, whereas the former corresponds to the fibril end assisting, through direct interaction, in the “correct misfolding” of the incoming monomer.

Different environmental conditions such as temperature, pH, salt components, shear forces, denaturants, concentration of protein, and co-solvents can lead to the formation of different amyloid strains (Table 1A and Figure 1A). Many *in vitro* (cross-)seeding experiments performed with a particular fibril strain, under conditions where a different strain would form *de novo*, confirmed the efficient imprinting of the conformational properties of the growing fibrils by the pre-formed fibrils, overcoming the structural preferences induced by the particular set of solution conditions (Table 1). The “conformational memory” of fibril strains used as seeds can in some cases be maintained over many generations of seeding (Jones and Surewicz, 2005; Frost et al., 2009).

**TABLE 1:** Overview over *in vitro* studies investigating the propagation of structural properties through (A) fibril elongation and (B) secondary nucleation.

**Protein**	**Condition varied to produce strains**	**Conditions of seed production**	**Observation and [Ref]**
**(A) Elongation**			
Insulin	Protein concentration	LCF (Low concentration fibrils), HCF (high concentration fibrils)	When LCF (HCF) used as seeds in the high (low) insulin concentration, the resulting fibrils have the structural properties of the seeds (Sakalauskas et al., 2019).
	Solvent composition	Ethanol, Water	Structural characteristics of fibrils completely dictated by the parent seed rather than the solvent conditions (Dzwolak et al., 2004, 2005).
	pH	pH 1.6–2	Conformational properties of the template were transferred to growing fibrils in spite of the unfavorable environmental condition (Sneideris et al., 2015a).
	Different sequences	Human insulin analog KR, bovine insulin (BI)	High concentrations of KR seeds revert the superstructural chirality of BI fibrils compared to the absence of KR seeds (Dzwolak et al., 2013).
			Homologous seeds have dominant effect on imprinting their conformational properties in daughter amyloid generations (Surmacz-Chwedoruk et al., 2014).
Glucagon	Solution condition	Salt concentration Protein concentration and different temperature	Some of the characteristics of strains may be propagated by seeding but other characteristics, e.g., thermostabilty, are not inherited upon seeding (Pedersen et al., 2006).
K3 fragment of (β2-microglob.	Different solution	F210, F218	The seeding of f201 monomer with f218 seeds lead to the formation of fibrils with f218 properties, but repeating the seeding reaction leads to gradual disappearance of f218 properties and f218 fibrils are transformed completely into f210 fibrils over several cycles (Yamaguchi et al., 2005).
Aβ	Sequences	Aβ1–40, Aβ1–42	Aβ1–42 can be cross-templated by Aβ1–40 fibril ends, while Aβ1–40 monomers are not efficiently incorporated into the end of Aβ1–42 fibrils (Brännström et al., 2018).
	Solution conditions	Quiescent or agitation condition	The results showed that quiescent and agitated parent fibrils show pronounced structural differences that are transmitted to subsequent generations of fibrils (Petkova et al., 2005).
α - synuclein	Solution conditions	Different buffers	Cross-seeding with α-syn fibril strains showed that these strains imprint their structural properties to soluble α-syn molecules upon their incorporation within fibrils (Bousset et al., 2013; Peduzzo et al., 2020).
PrP	Different sequence	Human PrP, Mouse PrP, Hamster PrP	Cross seeding of these variants leads to the formation of fibril strains that have properties like the parent fibril (Jones and Surewicz, 2005).
	Solution conditions	2M or 4M of GuHCl	Cross seeding by these two different types of seeds showed that conformational stability can be transmitted to the final fibrils in a seed concentration-dependent manner (Sneideris et al., 2015b).
Tau	Sequence	K18, K19	K18 monomers incorporate into K18 seeds but K19 monomers do not, while both K18 and K19 monomers can add onto K19 fibrils (Dinkel et al., 2011).
Sup35	Solution condition	Temperature (4 and 37°C)	Two strains of Sup 35 termed SC4 and SC37 have different division rates and conformational properties. These differences determine the acceptable conformation of SupNM monomers that could be incorporated at the end of these strains (Toyama et al., 2007).
			SC4 strain from *S. cerevisiae Sup35* can seed SupNM polymerization from *C. albicans* and imprints is properties onto the newly formed fibrils (Tanaka et al., 2005).
**(B) Secondary nucleation**			
Insulin	Protein concentration	0.2–1 mM	As seed concentration is decreased, propagation of seed structural properties decreases, as evaluated by AFM imaging and FTIR spectroscopy (Sakalauskas et al., 2019).
PrP	Denaturant concentration	2 and 4 M GndHCl	As seed concentration is decreased, propagation of seed structural properties decreases, as evaluated by AFM imaging, chemical depolymerisation and FTIR spectroscopy (Sneideris et al., 2015b).
α-synuclein	pH	pH 7 and pH 5	As seed concentration is decreased under conditions conducive for secondary nucleation (pH 5), propagation of seed structural properties decreases, as evaluated by AFM/TEM imaging and protease resistance (Peduzzo et al., 2020).
Aβ	Sequence	Aβ40 vs. Aβ42	Aβ42 fibrils formed through cross-surface nucleation on Aβ40 fibril surfaces display the seeding properties of de novo formed Aβ42 fibrils, as evaluated by SPR experiments (Brännström et al., 2018).
		Various point mutations	Cross-surface nucleation was found to be only efficient if monomer has same structural preference as seed fibril (Thacker et al., 2020).

## Secondary Nucleation and the Preservation of the Fibril Strain

Before being able to investigate whether or not secondary nucleation transmits the seed fibril strain, it first has to be established if a given amyloid fibril system is able to proliferate through secondary nucleation. In recent years, a range of amyloid proteins have been investigated in this respect and it has been found that secondary nucleation appears to be relatively widespread (Ruschak and Miranker, 2007; Foderà et al., 2008; Cohen et al., 2013; Buell et al., 2014; Lundqvist et al., 2021). Indeed, secondary nucleation might only be a special case of general surface nucleation (Buell, 2017). It has been shown that fibrils of one protein can nucleate from monomer on the surface of fibrils of another protein, while the same proteins are unable to elongate each others fibrils (Koloteva-Levine et al., 2020; Vaneyck et al., 2021). This difference suggests that cross-surface nucleation has much lower requirements for sequence similarity than cross-elongation.

Secondary nucleation can be either detected through systematic variation of monomer concentration in unseeded experiments, followed by kinetic analysis (Cohen et al., 2013), or else from the comparison of weakly seeded experiments with unseeded experiments under quiescent conditions (Buell et al., 2014). If the seed concentrations are too high (approx. ≥1% by mass and more) then the kinetic traces of the aggregation reaction are dominated by the growth of the seed fibrils (Buell, 2019) and any contribution from secondary nucleation can be very difficult to resolve. A systematic decrease in seed concentration will eventually reveal the presence, or not, of secondary nucleation (Buell et al., 2014; Sneideris et al., 2015b; Sakalauskas et al., 2019).

So far, compared to fibril elongation, only a small number of studies have addressed the question whether or not secondary nucleation propagates the structural information of the seed fibril strain. In the case of insulin, it was found that the degree of transmission of the seed strain characteristics decreases with decreasing seed concentration (Sakalauskas et al., 2019). This finding suggests that only in a seed concentration regime dominated by elongation, efficient propagation of structural properties takes place, whereas an increasing proportion of newly generated fibrils from secondary nucleation leads to a loss of strain propagation. Similar findings were made with a fragment of the mouse prion protein (Sneideris et al., 2015b), as well as with human α-synuclein (Peduzzo et al., 2020). In these studies, the different strains were distinguished by spectroscopy (Sneideris et al., 2015b; Sakalauskas et al., 2019), equilibrium depolymerisation (Sneideris et al., 2015b), as well as resistance to proteolytic degradation (Peduzzo et al., 2020) and high resolution imaging (Sakalauskas et al., 2019; Peduzzo et al., 2020). Apart from providing insight into fundamental aspects of secondary nucleation, these types of experiments also establish that secondary nucleation is clearly distinct from elongation (Scheidt et al., 2019; Peduzzo et al., 2020; Table 1B) provides a summary of these studies.

Similar experiments have also been performed for cases where two closely related sequences could be shown to form different fibril structures under a given set of solution conditions. In one such study, involving surface plasmon resonance (SPR) experiments with the Aβ 1-40 and Aβ 1-42 peptides the strain characteristics were evaluated based on the cross-seeding ability (Aβ 1-40 fibrils seed both types of monomer, but Aβ 1-42 fibrils are only capable of self-seeding). It was found that the structural characteristics of fibrils formed through cross-surface nucleation (the term secondary nucleation should be reserved to scenarios where the monomer and the seed fibril have the same amino acid sequence) were not defined by the seed structure, in contrast to fibril elongation. In another recent study also involving the Aβ peptide, several sequence variants were investigated. It was shown that surface cross-nucleation was only efficient if the monomer had an innate preference (as judged from the fibrils formed *de novo*) for the structure of the seed fibril (Thacker et al., 2020). While these experiments are suggestive of some degree of specificity of fibril surface nucleation, they do not demonstrate the actual transmission of the template structure.

In summary, therefore, we do not currently have any evidence for the direct transmission of structural properties of amyloid fibrils by secondary nucleation, and secondary nucleation appears merely as a particular example of the general phenomenon of surface-catalysed formation of amyloid fibrils.

## Potential Origin of the Differences in the Strain Propagation Properties Between Fibril Elongation and Secondary Nucleation

The existence of a multitude of fibril strains for most amyloid proteins, in the form of alternative structures formed by a given amino acid sequence, illustrates presumably the degeneracy of the amyloid free energy landscapes, i.e., the existence of several free energy minima with comparable stability. However, the relative populations of different strains in any given scenario *in vitro* or *in vivo* does not necessarily solely reflect the relative thermodynamic stabilities, as they can also be influenced by differences in nucleation kinetics. For example could different strains nucleate with different efficiencies on surfaces vs. in the bulk, or display different concentration dependencies of their nucleation rates. The nucleation of amyloid fibrils is associated with high free energy barriers (Buell, 2017; Cohen et al., 2018), possibly due to the large loss in entropy associated with the formation of a multimeric nucleus from two or more often disordered monomeric precursor molecules. The addition to a seed fibril enables the often highly flexible monomer to be efficiently templated into the same structure as the seed, even if this structure does not correspond to the most stable one under these conditions. The seeding effect only continues if the last added monomer adopts the correct structure. Otherwise, the incorrectly incorporated monomer will re-arrange or dissociate again. Such a selectivity for correctly added monomers does not seem to operate for monomers adsorbed to the fibril surface, the precursors of secondary nuclei (Šarić et al., 2016). The binding of monomer to fibril surfaces has been measured for the Aβ peptide and was found to be of two orders of magnitude lower affinity than that of correct incorporation into fibril ends (Šarić et al., 2016) and strongly exothermic (Cohen et al., 2018). These characteristics are reminiscent of the generic requirements of interaction between a catalyst and its substrate, which must be neither too strong, nor too weak (Sabatier principle; Kari et al., 2018).

In the case of α-synuclein, it has been shown that the binding of protein to the fibril surface is strongly enhanced at mildly acidic pH, where also secondary nucleation rates are very high. Therefore, secondary nucleation appears to correlate with a generic, non-specific attachment of monomer to the fibril surface. Nucleation is then presumably favored for the same reasons that binding to other types of interfaces accelerate it (Galvagnion et al., 2015; Buell, 2017). While being strongly favored through binding to the surface of the seed fibrils, nucleation on fibril surfaces appears to still be subject to the influence of the solution conditions, as in the case of nucleation on foreign surfaces, such as the polymer-water or air-water interfaces (Bousset et al., 2013; Campioni et al., 2014). The only existing evidence for nucleation on fibril surfaces to be different from generic, non-specific surface nucleation is the data on the Aβ peptide, which suggests that this type of nucleation is inefficient if the monomer has a different structural preference from the seed fibril (Brännström et al., 2018; Thacker et al., 2020).

Based on these existing data, it is too early to draw a final conclusion whether or not secondary nucleation can play a role in the propagation of strain homogeneity and in diseased organisms. Perhaps the physico-chemical environment *in vivo* allows only a limited number of strains to form *de novo*, and hence faithful strain transmission is not required in order to maintain strain homogeneity. There is, however, recent evidence that points toward the possibility that in cases where strain propagation is observed, most notably the mammalian prions, fragmentation could be the mechanism responsible for this propagation (Meisl et al., 2021). More research is needed to establish the degree of strain homogeneity in additional *in vivo* settings and to investigate the strain propagation of secondary nucleation for additional proteins and solution conditions that mimic *in vivo* conditions as closely as possible. The observation that only a fraction of the oligomers generated by secondary nucleation ultimately converts into fibrils (Michaels et al., 2020) also suggest the possibility that the roles of secondary nucleation in the generation of toxic species and in the overall propagation of the pathology could be de-coupled to some extent.

## Outlook

We believe that the future of this line of research lies in the combination of various cross-seeding schemes, between fibrils of the same sequence formed under different conditions, as well as of different sequences under the same conditions, with high resolution cryo-EM. This combination of methods will ultimately reveal exactly how fibril elongation is able to propagate structural information, and whether in secondary nucleation the properties of the newly generated fibrils are solely defined by the solution conditions.

## Author Contributions

AB conceived the study. AB and SH wrote the manuscript. AP contributed to the initial draft of the manuscript and commented on the final manuscript. All authors contributed to the article and approved the submitted version.

## Conflict of Interest

The authors declare that the research was conducted in the absence of any commercial or financial relationships that could be construed as a potential conflict of interest.
